# Adsorption of a synthetic TLR7/8 ligand to aluminum oxyhydroxide for enhanced vaccine adjuvant activity: A formulation approach

**DOI:** 10.1016/j.jconrel.2016.11.011

**Published:** 2016-12-28

**Authors:** Christopher B. Fox, Mark T. Orr, Neal Van Hoeven, Sarah C. Parker, Traci J.T. Mikasa, Tony Phan, Elyse A. Beebe, Ghislain I. Nana, Sharvari W Joshi, Mark A. Tomai, James Elvecrog, Timothy R. Fouts, Steven G. Reed

**Affiliations:** a IDRI, 1616 Eastlake Ave, Seattle, WA 98102, USA; b Dept of Global Health, University of Washington, Seattle, WA 98104, USA; c 3M Drug Delivery Systems, 3M Center, 275-3E-10, St. Paul, MN 55144, USA; d Profectus Biosciences, Inc., Baltimore, MD 21224, USA

## Abstract

For nearly a century, aluminum salts have been the most widely used vaccine adjuvant formulation, and have thus established a history of safety and efficacy. Nevertheless, for extremely challenging disease targets such as tuberculosis or HIV, the adjuvant activity of aluminum salts may not be potent enough to achieve protective efficacy. Adsorption of TLR ligands to aluminum salts facilitates enhanced adjuvant activity, such as in the human papilloma virus vaccine Cervarix®. However, some TLR ligands such as TLR7/8 agonist imidazoquinolines do not efficiently adsorb to aluminum salts. The present report describes a formulation approach to solving this challenge by developing a lipid-based nanosuspension of a synthetic TLR7/8 ligand (3M-052) that facilitates adsorption to aluminum oxyhydroxide via the structural properties of the helper lipid employed. In immunized mice, the aluminum oxyhydroxide-adsorbed formulation of 3M-052 enhanced antibody and TH1-type cellular immune responses to vaccine antigens for tuberculosis and HIV.

## Graphical abstract

**Figure:**
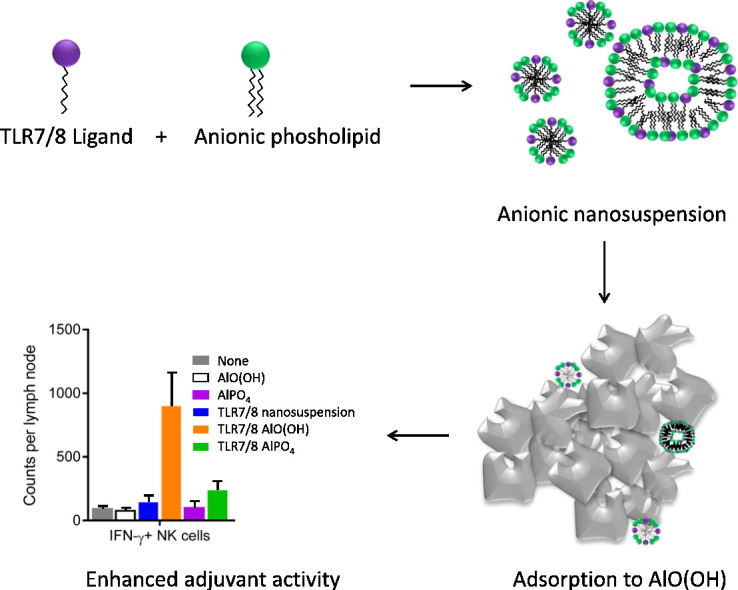

## 1 Introduction

Since Glenny's pioneering work in the early 20th century [1], aluminum salts have become the most widely used adjuvants in human vaccines, generating an unrivalled history of safety and suitability with various vaccine antigens. Aluminum salts generally consist of semi-crystalline nano- and micro-particles with a large surface area and a high charge density. They may be most effective as adjuvants when vaccine antigens are optimally adsorbed to the surface of the aluminum salt particles [2]. Aluminum salts are effective in boosting antibody responses to vaccine antigens, but there is little indication that they substantially augment cellular immunity to vaccine antigens. Induction of effective cellular immunity is likely essential for developing effective vaccines for several diseases including tuberculosis, HIV, and malaria. Therefore, the adsorption of additional immunostimulants to aluminum salts should also be a paramount consideration in vaccine formulation development. Thus, an important advancement in the clinical use of adjuvants occurred in 2009 when the US FDA approved GlaxoSmithKline's human papilloma virus vaccine Cervarix® for human use in 2009; Cervarix® contains AS04, an adjuvant system consisting of the Toll-like receptor 4 (TLR4) ligand monophosphoryl lipid A (MPL®) adsorbed to aluminum oxyhydroxide, resulting in potent adjuvant activity [3].

Combining TLR ligands with aluminum salts is an attractive approach given that aluminum salts have an established safety and manufacturability history, are familiar to regulatory agencies, and are more amenable to a stable single vial liquid presentation that can promote Th1-type immunity when combined with a TLR ligand [4]. Moreover, adsorption to aluminum of TLR ligands co-localizes antigen and adjuvant, and facilitates reduction of antigen and/or TLR ligand dose [5], [6], [7]. Therefore it is of high interest to develop aluminum salt-based formulations of other pattern recognition receptor (PRR) ligands (besides TLR4 agonists) to enhance antigen-specific Th1-type immunogenicity and protective efficacy [5], [8], [9]. Some PRR ligands, such as the TLR4 ligand MPL® and the TLR9 ligand CpG oligonucleotides, adsorb to some aluminum salts due to physicochemical structure compatibility. For example, aluminum oxyhydroxide is positively charged and adsorbs antigens or TLR ligands due to phosphate ligand exchange and/or electrostatic interactions [2], [10]. In contrast to the TLR4 ligands (which often contain phosphate groups) or nucleotide-based TLR9 and TLR3 ligands (which are negatively charged), other PRR ligands of interest including the TLR7/8 agonist imidazoquinolines may not contain structural moieties, such as phosphate groups or anionic charge, that would promote adsorption to aluminum oxyhydroxide. The situation is further complicated for insoluble lipid-based PRR ligands, which must first be formulated into aqueous suspensions prior to adsorption to aluminum salt [10].

In earlier work, we developed an aqueous nanosuspension of an insoluble synthetic TLR4 ligand (GLA) by formulating with a phosphatidylcholine to form GLA-AF, which was shown to adsorb to aluminum oxyhydroxide through phosphate ligand exchange [10]. Characterization of the adsorption interactions between aluminum oxyhydroxide and the phospholipid excipient component of GLA-AF led us to hypothesize that helper lipids could promote the adsorption of insoluble PRR ligands to aluminum oxyhydroxide even if the PRR ligand does not contain a phosphate or other anionic group. Moreover, the versatility of this approach could allow adsorption of the same PRR ligand to different types of aluminum salts depending on the structure of the helper lipid with which it is complexed. We have selected an insoluble TLR 7/8 ligand that does not contain a phosphate or other anionic group to demonstrate this approach.

**Fig. 1:**
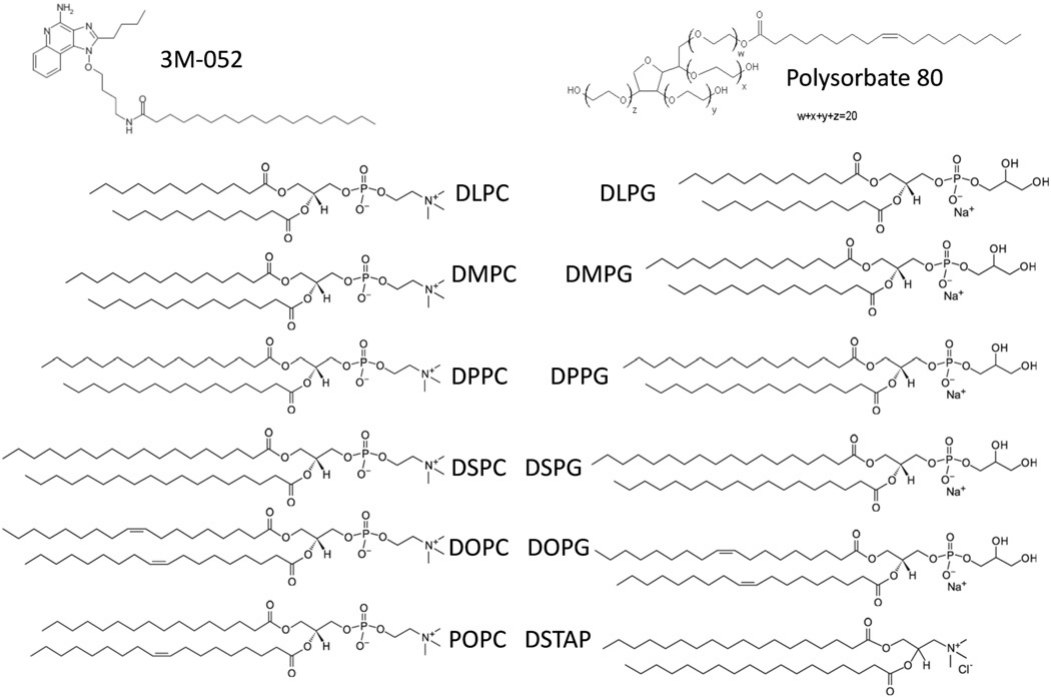
Chemical structure of 3M-052 and screened helper lipids.

Appropriate formulation of TLR7/8 agonists is an attractive adjuvant development approach for several reasons including manufacturability, induction of potent TH1 responses, and prior use in an FDA-approved product. The ability of imidazoquinolines to target TLR7 and/or TLR8 to generate enhanced TH1-type innate immune responses, including IgG2 antibodies in mice, has been documented in the literature [11], [12], [13]. As synthetic small molecules, imidazoquinolines can be manufactured cost effectively and at high purity. The TLR7 ligand imiquimod is the active component in the topical cream Aldara®, approved for human immunotherapeutic use to treat skin cancer and genital warts. However, injected imidazoquinolines as vaccine adjuvants have not progressed beyond early phase clinical testing. Due to their small size, it is hypothesized that soluble unformulated imidazoquinolines such as R848 rapidly diffuse from the injection site, causing systemic immune activation rather than localized stimulation. For this reason, strategies to “slow down” imidazoquinoline diffusion such as covalent conjugation to vaccine antigens or encapsulation in particulate formulations have shown promise in preclinical testing [8], [14], [15], [16], [17]. Smirnov et al. describe a chemical synthesis approach in which the addition of an 18-carbon chain to an imidazoquinoline structure maintains local adjuvant activity but does not generate the systemic responses evident with non-lipidated structures such as R848 [16]. This new molecule, called 3M-052 (Fig. 1), is thus more amenable to incorporation in lipid-based formulations such as nanosuspensions, liposomes, or emulsions. In the present work, we describe a formulation approach involving the development of nanosuspensions involving helper lipids with phosphate or other charged groups to modulate the adsorption interactions between the synthetic insoluble TLR7/8 ligand 3M-052 [16] and aluminum salts in order to create a new vaccine adjuvant formulation that enhances antibody and cellular immunogenicity to co-adsorbed recombinant tuberculosis or HIV vaccine antigens.

## 2 Materials and methods

### 2.1 Adjuvant formulation materials

Synthetic 1,2-dilauroyl-sn-glycero-3-phosphcocholine (DLPC), 1,2-dimyristoyl-sn-glycero-3-phosphocholine (DMPC), 1,2-dipalmitoyl-sn-glycero-3-phosphocholine (DPPC), 1,2-distearoyl-sn-glycero-3-phosphocholine (DSPC), 1,2-dioleoyl-sn-glycero-3-phosphocholine (DOPC), 1,2-dilauroyl-sn-glycero-3-phospho-(1′-rac-glycerol) (DLPG), 1,2-dimyrsitoyl-sn-glycero-3-phospho-(1′-rac-glycerol) (DMPG), 1,2-dipalmitoyl-sn-glycero-3-phospho-(1′-rac-glycerol) (DPPG), 1,2-distearoyl-sn-glycero-3-phospho-(1′-rac-glycerol) (DSPG), 1,2-dioleoyl-sn-glycero-3-phospho-(1′-rac-glycerol) (DOPG), 1,2-distearoyl-3-trimethylammonium-propane (DSTAP), 1,2-dipalmitoyl-3-trimethylammonium-propane (DPTAP), and glucopyranosyl lipid adjuvant (GLA, also known as PHAD®) were purchased from Avanti Polar Lipids Inc. (Alabaster, AL). Polysorbate 80 was purchased from J.T. Baker (San Francisco, CA). Poloxamer 188 was purchased from Spectrum Chemical (Gardena, CA). Saline solution (0.9% w/v) was purchased from Teknova (Hollister, CA). CpG ODN was obtained from Avecia (Milfrod, MA). Alhydrogel® ‘85’ and AdjuPhos® were purchased from E.M. Sergeant Pulp & Chemical Co. (Clifton, NJ). 3M-052 (S-36862) was synthesized by 3M Drug Delivery Systems. The chemical structure has been previously disclosed [16].

### 2.2 Adjuvant formulation manufacture

Aqueous nanosuspensions were manufactured by dispersing 3M-052 or GLA with each lipid excipient (see Fig. 1) at a 1:2 M ratio (adjuvant:lipid), in chloroform or a mixture of chloroform, methanol, and water. The solvent was then evaporated using a Genevac EZ-2 centrifugal evaporator (Stone Ridge, NY) for several hours or overnight. The dried films were rehydrated in ultrapure water, then sonicated in a Crest powersonic CP230D (Trenton, NJ) sonicating water bath at ~ 60 °C for up to several hours or until the formulations were translucent with no visible particles. To formulate alum-containing compositions, the aqueous nanosuspensions were mixed with Alhydrogel® or AdjuPhos®. For the immunogenicity studies, recombinant vaccine antigens (ID93 for tuberculosis or FLSC for HIV) were mixed together with the nanosuspension, alum, and the indicated diluent to deliver an antigen dose of 0.5 μg or 10 μg, a 3M-052 dose of 0.1–10 μg, and an aluminum dose of 100 μg, in a 100-μl total volume as indicated in the figure captions. ID93 is a recombinant fusion protein containing four tuberculosis antigens [18]. FLSC is a recombinant antigen containing a full-length single-chain gp120-CD4 complex [19].

### 2.3 Adjuvant formulation characterization and stability

Aqueous nanosuspensions were characterized for particle size by dynamic light scattering (DLS) using the Malvern Instruments (Worcestershire, UK) Zetasizer Nano-S or –ZS. Aqueous nanosuspensions were diluted 1:10 or 1:100 fold in water in a polystyrene cuvette prior to analysis, which consisted of three measurements resulting in a scattering intensity-biased average diameter value reported as the *Z*-ave. Zeta potential was measured using the Zetasizer Nano-ZS using a disposable capillary cell, with nine consecutive measurements collected from each sample prepared at 1:10 dilution in water. In general, 3M-052 concentration was measured by UV absorbance at 322.5 nm after diluting formulations 1:20 into ethanol:HCl (98:2 v:v) and comparing to a standard curve. The dilution into organic solvent removes potential interference from light scattering of the nanosuspension particles. However, for the 3M-052 binding isotherm, where maximum sensitivity was desired, no dilution was performed for the samples or the standards. CpG concentration was measured by UV absorbance at 260 nm after 1:20 dilution into ethanol:HCl. GLA concentration was measured by reverse-phase HPLC with an C18 column (Atlantis T3 or Agilent XBridge) and charged aerosol detection (CAD) using a methanol:chloroform:water mobile gradient as described previously [20]. To detect unbound TLR ligand, alum-containing formulations were centrifuged briefly as indicated and the supernatant assayed by UV absorbance or HPLC-CAD. Unless otherwise noted, centrifugation time was 5 min at 16,000 ×* g*.

### 2.4 Cryo-transmission electron microscopy (cryoTEM) imaging

Samples were preserved in vitrified ice supported by holey carbon films on 400-mesh copper grids. Samples were prepared by applying a 3 μl drop of sample suspension to a cleaned grid, blotting away with filter paper, and immediately proceeding with vitrification in liquid ethane. Grids were stored under liquid nitrogen until transferred to the electron microscope for imaging. Electron microscopy was performed using an FEI Tecnai T12 electron microscope, operating at 120 keV equipped with an FEI Eagle 4 k × 4 k CCD camera. Vitreous ice grids were transferred into the electron microscope using a cryostage that maintains the grids at a temperature below − 170 °C. Images of each grid were acquired at multiple scales to assess the overall distribution of the specimen. After identifying potentially suitable target areas for imaging at lower magnifications, high magnification images were acquired at nominal magnifications of 110,000 × (0.10 nm/pixel), 52,000 × (0.21 nm/pixel) and 21,000 × (0.50 nm/pixel). The images were acquired at a nominal underfocus of –2 μm (110,000 ×), − 3 μm to –2 μm (52,000 ×) and –5 μm (21,000 ×) and electron doses of ~ 9–42 e/Å^2^.

### 2.5 Antigen adsorption to aluminum salts

Binding efficiency of 3M-052 and ID93 or FLSC to Alhydrogel® and AdjuPhos® was determined by UV–Vis Spectroscopy and SDS-PAGE with silver stain. 1-mL of formulation was prepared by mixing saline diluent, antigen, 3M-052-AF (aqueous nanosuspension of 3M-052 and DSPG), and/or aluminum salt. To determine FLSC adsorption, 30 μl of sample supernatant was mixed with 10 μl of 4 × reducing or non-reducing LDS Sample Buffer, following which 20–25 μl was loaded into a 10-lane SDS-PAGE gel with 15 μl of SeeBlue2 Prestained Standard. To determine ID93 adsorption, 45 μl of sample supernatant was mixed with 15 μl of 4 × reducing LDS Sample Buffer, following which 25 μl was loaded into a 10-lane SDS-PAGE gel with 15 μl of SeeBlue2 Prestained Standard. The gels were run for 55 min at 190 V and then placed into a fixing solution of 50:40:10 EtOH:CH_3_COOH:H_2_O for overnight. The gels were then stained according to the directions provided by Sigma-Aldrich (Saint Louis, MO) ProteoSilver Plus Silver Stain Kit.

### 2.6 Animals and immunizations

C57Bl/6 and B6.129S1-TLR7^tm1Flv^/J (TLR7^-/-^) were purchased from Jackson Laboratories (Bar Harbor, ME). Mice were immunized by intramuscular injection with the recombinant TB vaccine antigen ID93 (0.5 μg/dose) or the HIV vaccine antigen FSLC1 (10 μg/dose) adjuvanted with AdjuPhos, Alhydrogel®, 3M-052 + Alhydrogel®, 3M-052 + AdjuPhos, or GLA + Alhydrogel®. The final adjuvant dose was 5 μg GLA or 0.1–10 μg 3M-052 with 200 μg of AdjuPhos or Alhydrogel® in 100 μl. Mice were boosted three weeks after the first immunization. All mice were maintained in specific pathogen-free conditions. All procedures were approved by the IDRI Institutional Animal Care and Use Committee.

### 2.7 Antibody titers

Mouse sera (*N* = 5/group) were prepared 21 days after immunization by collection of retro-orbital blood into microtainer serum collection tubes (VWR International, West Chester, PA), followed by centrifugation. Each serum sample was then analyzed by antibody capture ELISA. Briefly, ELISA plates (Nunc, Rochester, NY) were coated with 2 μg/ml of the immunizing antigen in 0.1 M bicarbonate buffer and blocked with 1% BSA-PBS. Then, in consecutive order and following washes in PBS/Tween20, serially diluted serum samples, anti-mouse IgG, IgG1 or IgG2c-HRP (Southern Biotech, Birmingham, AL) and ABTS-H2O2 (Kirkegaard and Perry Laboratories, Gaithersburg, MD) were added to the plates. Plates were analyzed at 405 nm (ELX808, Bio-Tek Instruments Inc., Winooski, VT). Endpoint titers were calculated using Prism software V6 (GraphPad). Alternatively vaginal lavage fluid was collected three weeks after the third immunization with FLSC and analyzed for antibody titers using the same methods.

### 2.8 Intracellular cytokine staining

One week after the final immunization splenocytes were isolated. Red blood cells were lysed using Red Blood Cell Lysis Buffer (eBioscience) and resuspended in RPMI 1640 and 10% fetal bovine serum (FBS). Cells were plated at 2 × 10^6^ cells/well in 96-well plates and were stimulated for 2 h with the immunizing antigen (10 μg/ml), or unstimulated at 37 °C. GolgiPlug (BD Biosciences) was added and the cells were incubated for an additional 8 h at 37 °C. Cells were washed and surface stained with fluorochrome-labeled antibodies to CD4 (clone GK1.5), CD44 (clone IM7) and CD8 (clone 53-6.7) (BioLegend and eBioscience) in the presence of anti-CD16/32 (clone 2.4G2) for 20 min. Cells were washed and permeabilized with Cytofix/Cytoperm (BD Biosciences) for 20 min. Cells were washed twice with Perm/Wash (BD Biosciences) and stained intracellularly with fluorochrome-labeled antibodies to CD154 (clone MR1), IFN-γ (clone XMG-1.2), IL-2 (clone JES6-5H4), TNF (clone MP6-XT22), GM-CSF (clone MP1-22E9), IL-5 (clone TRFK5), and IL-17A (clone TC11-18H10.1) (BioLegend and eBioscience) for 20 min at room temperature. Cells were washed and resuspended in PBS. Up to 10^6^ events were collected on an LSRFortessa flow cytometer (BD Biosciences). Data were analyzed with FlowJo (TreeStar). Cells were gated as singlets > lymphocytes > CD4 + CD8– > cytokine positive or CD44^hi^ > cytokine positive. In the case of ID93, antigen-specific response frequencies were determined by subtracting the frequency of response positives of unstimulated cells from antigen stimulated cells.

### 2.9 Antibody secreting cell ELISPOT assay

Antigen-specific antibody secreting cells present in the bone marrow were quantified using an ELISPOT assay. One day prior to assay initiation, Multiscreen ELISPOT plates (Millipore) were coated with 1μg of antigen/well, and incubated overnight. Blocked plates were washed three times with washing buffer (PBS + 0.5% Tween 20), blocked with collection medium for 2 h, and washed 3 times. Bone marrow was collected 21 days post-immunization in RPMI medium supplemented with 10% FBS, quantified using a Guava automated cell counter (Millipore) and resuspended to 1 × 10^6^ cells/mL. Cells were serially diluted 3-fold, added to plates, and incubated for 5 h at 37 °C. Secreted antibody was detected by addition of a 1:100 dilution of horse radish peroxidase (HRP) conjugated goat anti-mouse IgG antibody (Southern Biotech). Spots were visualized with AEC Peroxidase substrate kit (Vector Labs) according to manufacturer's instructions. Spots were quantitated on a CTL bioanalyzer.

### 2.10 Innate immune response

18 h after intramuscular immunization into the gastrocnemius muscle the draining popliteal lymph node was collected and dissociated in PBS containing protease inhibitors (Thermo Fisher Scientific). Cells were surface stained for CD8, CD90.2 (clone 53-2.1), CD19 (clone 1D3), NK1.1 (clone PK136), CD11c (clone N418), CD11b (clone M1/70), Ly6G (clone 1A8), Ly6C (HK1.4), CD69 (clone H1·2F3), and CD86 (clone GL1) for 20 min on ice. Cells were washed and permeabilized with Cytofix/Cytoperm (BD Biosciences) for 20 min. Cells were washed twice with Perm/Wash (BD Biosciences) and stained intracellularly with fluorochrome-labeled antibodies to IFN-γ and proIL-1β (clone NJTEN3) (BioLegend and eBioscience) for 20 min at room temperature. Cells were washed and resuspended in PBS. Up to 10^6^ events were collected on an LSRFortessa flow cytometer (BD Biosciences). Data were analyzed with FlowJo (TreeStar). Cells were gated as singlets > cells > CD19 + CD90.2– (B cells), CD8 + CD90.2 + (CD8 T cells), CD8– CD90.2 + (CD4 T cells), CD8– CD19– NK1.1 + (NK cells), CD8– CD19– CD11c + (DCs), CD8–CD19– CD11b + Ly6C + (inflammatory monocytes), or CD19–CD11b + Ly6G + (PMNs).

### 2.11 Statistical analysis

Antibody and T-cell responses were analyzed by two-way ANOVA with Tukey's multiple comparison correction using Prism version 5 or later (GraphPad). Comparisons resulting in *p*-values < 0.05 were considered significant. Only meaningful comparisons are reported in the figures (adjuvanted groups vs. antigen alone; 3M-052-Alhydrogel® vs. Alhydrogel®, 3M-052-AF, or 3M-052-AdjuPhos®; 3M-052-AdjuPhos vs. AdjuPhos® or 3M-052-AF).

## 3 Results and discussion

### 3.1 Formulation development and physicochemical characterization

**Fig. 2:**
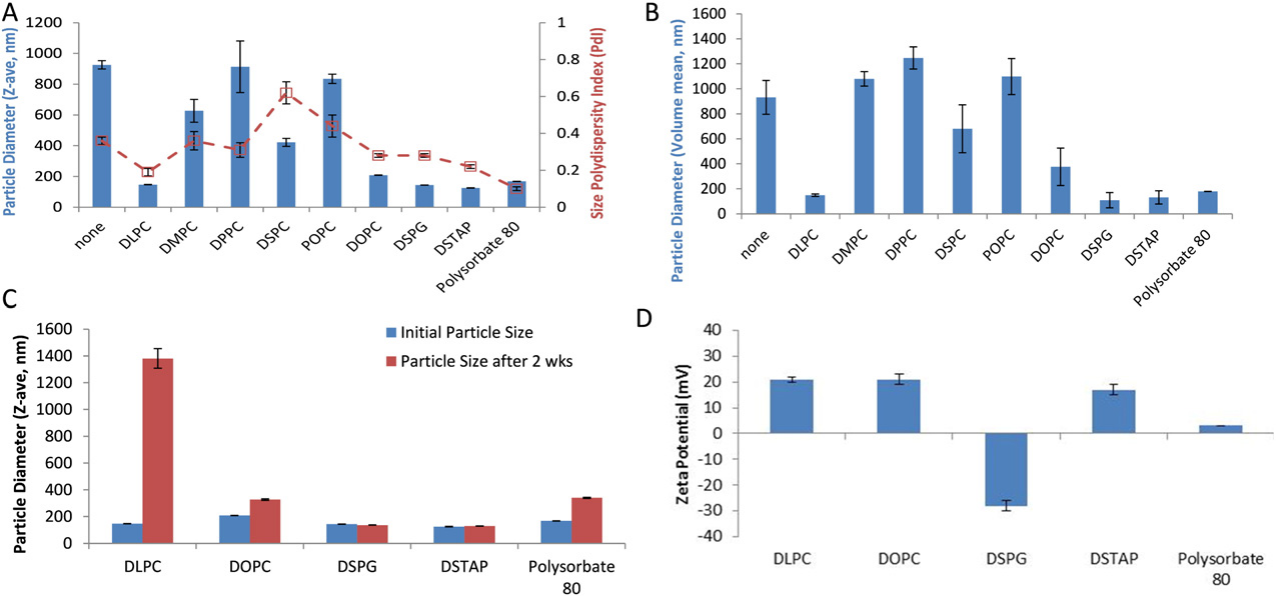
Physical properties of aqueous suspensions of 3M-052 and adsorption to aluminum salts. (a) Light scattering intensity-based particle size (*Z*-ave) and polydispersity index of 3M-052 aqueous suspensions at time of manufacture (shown is average ± s.d. of three measurements from same sample). (b) Volume-based mean particle size of the same samples based on mathematical conversion of light scattering intensity. (c) Light scattering intensity-based particle size stability over 2 weeks for selected 3M-052 suspensions stored at 5 deg C (shown is average ± s.d. of three measurements from same sample). (d) Zeta potential of selected 3M-052 suspensions (shown is average ± s.d. of nine measurements from the same sample for zeta potential).

Aqueous nanosuspensions of TLR ligands are formed by adding a suitable helper lipid or surfactant and inputting energy (e.g. sonication) to break down the particle size of the lipid complex [21]. To determine the most suitable helper lipid to form nanosize particles with the insoluble TLR7/8 ligand 3M-052, we screened a range of phospholipids and surfactants (Fig. 1). These helper lipids were first mixed with 3M-052 in organic solvent at a molar ratio of 1:2 (3M-052:helper lipid), based on our previous work with a TLR4 ligand [10]. Following evaporation of the solvent, the formulations were hydrated and sonicated to reduce particle size. Sonication of 3M-052 without helper lipid generated a large and heterogeneous particle size, and thus 3M-052 alone was not employed in further physicochemical or biological studies. Several formulations demonstrated acceptable particle size (< 200 nm to enable potential for terminal sterile filtration) after manufacture as determined by dynamic light scattering (Fig. 2A–B) and were selected for further study. The nature of the helper lipid or surfactant dictated formulation particle size and physical stability. Some formulations had grown significantly in size (DLPC, DOPC, polysorbate 80) indicating formulation physical instability while others demonstrated little change (DSPG, DSTAP) upon storage at 5 °C for 2 weeks (Fig. 2C). Most of the formulations were positively charged (Fig. 2D), as expected due to the chemical structure of imdazoquinolines such as 3M-052, which have a pKa ~ 7 [22]. However, DSPG caused formation of anionic particles due to the negative charge of the phosphate group in DSPG. An anionic aqueous suspension is of particular interest for vaccine adjuvant development due to the potential for adsorption to aluminum oxyhydroxide, which is the generally preferred aluminum salt for anionic recombinant protein antigens such as those employed in the present work. The DSPG-based nanosuspension of 3M-052, hereafter denoted 3M-052-AF, was physically stable for at least 6 months at 4 °C, showing little change in average particle size or particle size polydispersity (Fig. S1). Additional studies showed that phospholipids with the same headgroup as DSPG but different acyl chain length or saturation (Fig. 1) also promoted nanosuspension formation and adsorption of 3M-052 to aluminum oxyhydroxide (Table S1). DSPG was selected for further study since its acyl chain structure (saturated 18-carbon chain) matches that of 3M-052, although other PG-based lipids may be suitable for nanosuspension formation.

**Fig. 3:**
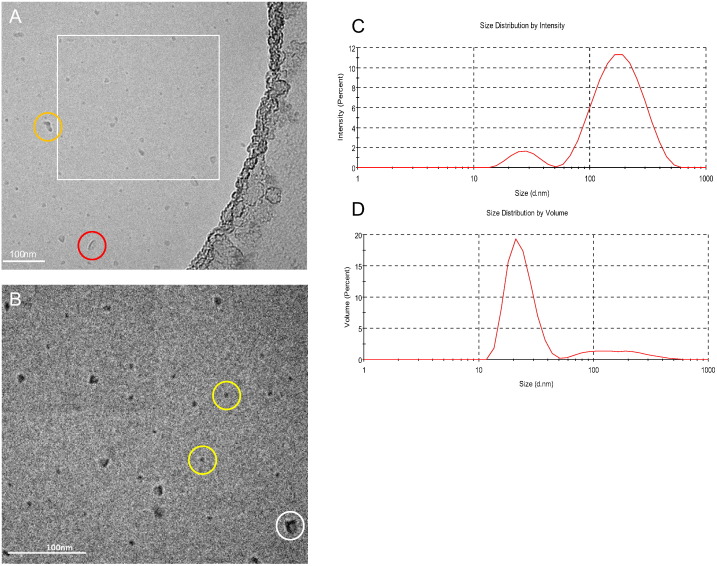
Particle size characteristics and morphology of 3M-052-AF. (A,B) CryoTEM images at two magnifications where the image shown in B (110,000 ×) represents the boxed area in A (52,000 ×); micelle-type structures are shown by the yellow circles; larger angular structures are indicated by the white circle; vesicle-type structures are indicated by the red circle; and particle aggregates are represented by the orange circle, scale bar = 100 nm. (C) intensity-based light scattering size distribution, (D) volume-based light scattering size distribution.

The morphology of 3M-052-AF as characterized by cryoTEM indicates a large percentage of micellar structures ~ 5–15 nm in diameter, although larger irregularly shaped particles and vesicles were also present (Fig. 3A–B). These size characteristics may appear to contradict the dynamic light scattering indicated above, where *Z*-ave values were generally over 100 nm. However, the light scattering intensity-based Z-ave value reported by dynamic light scattering is heavily influenced by even a small proportion of large particles since they scatter more light than smaller particles (light scattering is proportional to 10^6^ of the particle diameter) as evident in Fig. 3C. Mathematical conversion of the intensity based size distribution to a volume-based size distribution indicates many more particles in the ~ 20 nm range, although even volume-based size distributions are also skewed by larger particles, with a proportionality of 10^3^ (Fig. 3D). Nevertheless, the volume-based size distribution of 3M-052-AF is more consistent with the cryoTEM results (Figs. 3D and 2B).

**Fig. 4:**
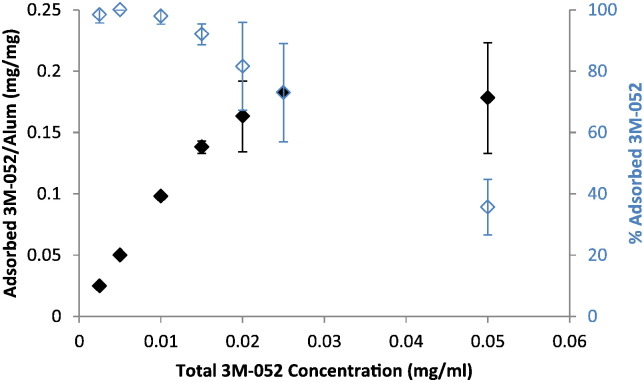
Adsorption isotherm of 3M-052-AF to Alhydrogel® as measured by UV absorbance of supernatant. Error bars represent standard deviation from three separate experiments using different batches of 3M-052-AF, where each sample preparation from each experiment was performed in duplicate.

**Fig. 5:**
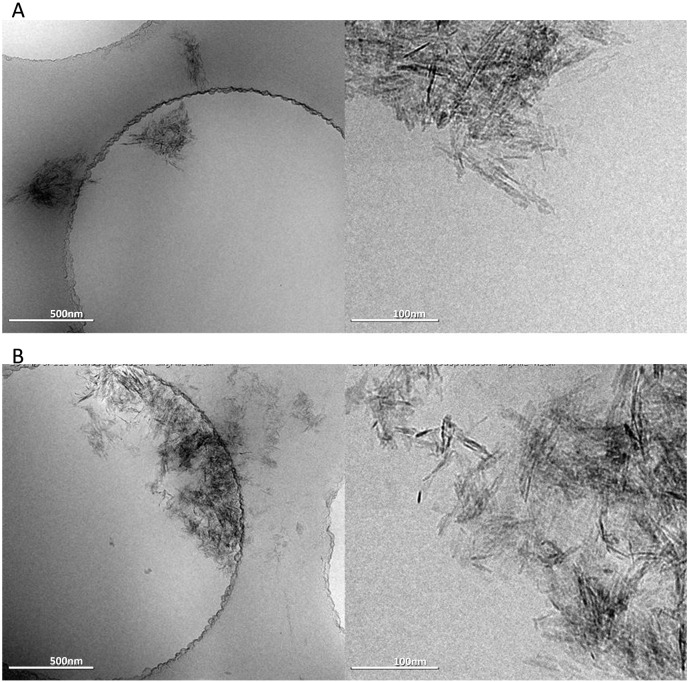
Aluminum oxyhydroxide morphology (with or without 3M-052-AF). (A) cryo-TEM image of aluminum oxyhydroxide particles, and (B) cryo-TEM image of aluminum oxyhydroxide particles with 3M-052-AF. Scale bar = 500 nm (left images) or 100 nm (right images).

To determine the adsorption capacity of Alhydrogel® for 3M-052-AF, the nanosuspension at various concentrations was mixed with aluminum oxyhydroxide and allowed to sit for ~ 30 min with intermittent vortexing, followed by centrifugation such that the aluminum particles pelleted. The supernatant was then assayed for 3M-052 to detect unbound material (Fig. 4). The adsorption capacity of the nanosuspension was ~ 0.18 mg per mg of aluminum. The doses of 3M-052 employed in the subsequent mouse immunogenicity experiments described in this work were well below 0.18 mg 3M-052 per mg of aluminum. Due to the small particle size of the 3M-052-AF, the nanosuspension particles were not evident in the cryoTEM images containing aluminum oxyhydroxide (Fig. 5A–B). Indeed, the morphology of the aluminum oxyhydroxide particles appeared much the same regardless of whether 3M-052-AF was present, with the notable exception that the crystalline aggregates appeared larger in size in the sample containing 3M-052-AF compared to the aluminum oxyhydroxide control.

The adsorption stability of 3M-052-AF on Alhydrogel® over time was evaluated by assaying samples for unbound 3M-052-AF before and after storage at 5 °C for 16 wks. Although there appeared to be partial loss of 3M-052-AF in the supernatants of the controls, there was no increase in detectable 3M-052-AF in the supernatants of Alum-containing samples, indicating no desorption over 16 wks (Table S2). Moreover, the presence of other TLR ligands did not appear to interfere with the adsorption of 3M-052-AF to aluminum oxyhydroxide, indicating that an Alum-based formulation containing multiple adsorbed PRR ligands could be feasible (Table S2). In contrast to aluminum oxyhydroxide, we did not observe binding of 3M-052-AF to aluminum phosphate.

To determine whether 3M-052-AF adsorbed to aluminum oxyhydroxide via a ligand exchange or electrostatic mechanism we evaluated the effect of ionic strength on adsorption, which neutralizes electrostatic mediated binding [23]. A trend of decreasing 3M-052-AF content in the supernatant of the control samples with increasing sodium chloride concentration is attributable to a rapid increase in particle size of 3M-052-AF upon exposure to saline, resulting in pelleting of the nanosuspension even when the aluminum was not present (Table S3). Nevertheless, increasing sodium chloride concentration did not appear to reduce the binding of 3M-052-AF to aluminum oxyhydroxide, indicating a ligand exchange mechanism rather than an electrostatic mechanism. Thus, by appropriate selection of helper lipid, the adsorption of 3M-052-AF to aluminum oxyhydroxide was facilitated.

Recent seminal work by Wu et al. demonstrated that chemical synthesis of new TLR7 ligands with phosphonate groups facilitated adsorption to aluminum oxyhydroxide, resulting in improved transient localized adjuvant activity while reducing systemic activation [8]. Alum-adsorbed TLR7 formulations effectively boosted antibody magnitude and quality to various vaccine antigens, including enhanced protection against challenge compared to the antigen with Alum alone or the TLR7 ligand alone [8]. In contrast, the formulation approach described herein does not require phosphonate groups on the PRR ligand to facilitate adsorption of PRR ligands to aluminum salts, and thus may have broader applicability. The ability to promote adsorption of PRR ligands to aluminum salts could provide a significant development advantage from a regulatory standpoint, since an alum-adsorbed PRR ligand formulation is already contained in approved vaccines such as Cervarix®. Importantly, a formulation-based approach could avoid the need to chemically modify existing agonist structures, relying instead on the properties of the formulation to promote adsorption to aluminum salts. Moreover, lipid formulation modifications could tailor PRR ligand adsorption preference to specific aluminum salts such that vaccine antigen and PRR ligand could be adsorbed to the same type of aluminum salt. Such excipient properties include length and saturation of acyl chains and headgroup structure/charge. We found that the latter appeared to be the main determinant in the ability of the nanosuspension to adsorb to aluminum salts, although acyl chain structure should also be taken into account in order to ensure stable suspension formation between PRR ligand and helper lipid.

### 3.2 Adjuvant biological activity

**Fig. 6:**
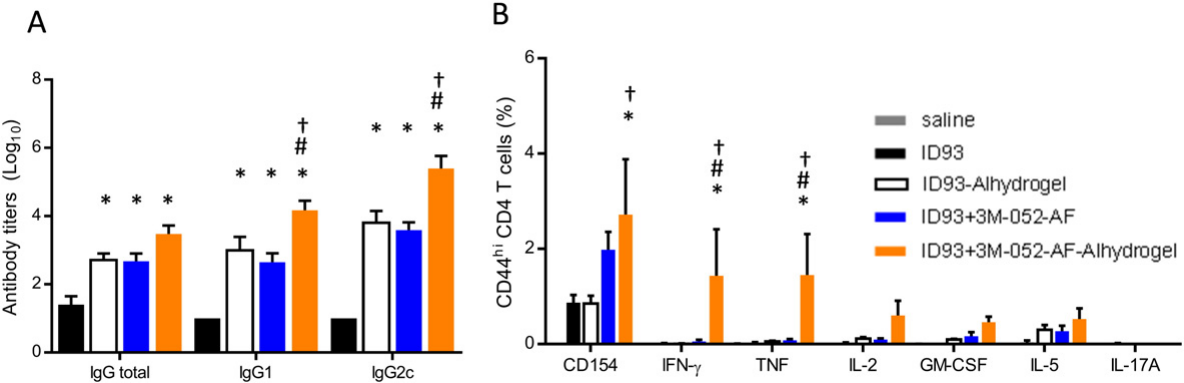
Alhydrogel® and 3M052-AF induce synergistic antigen-specific immunogenicity. C57BL/6 mice were immunized three times, three weeks apart via intramuscular injection with ID93 (0.5 μg) alone or adjuvanted with 3M-052-AF (0.5 μg), Alhydrogel®, or 3M-052-AF (0.5 μg) bound to Alhydrogel®. (A) Three weeks after the first immunization ID93-specific IgG1, IgG2c, and total IgG (IgGT) serum endpoint titers were determined by ELISA. *N* = 4–5 mice/group. (B) Four weeks after the final immunization splenocytes were restimulated with media or ID93 in the presence of Brefeldin A for 8 h and the frequency of cytokine producing CD4 T cells was determined by subtracting the media response from the ID93-specific response. Data are representative of two experiments with similar results with 4–5 animals per group. Mean ± s.e.m. is shown. *p < 0.05 vs. ID93, #*p* < 0.05 vs. ID93 + 3M-052-AF, †*p* < 0.05 vs. ID93-Alhydrogel®.

To determine whether the binding of 3M-052-AF to aluminum oxyhydroxide alters the in vivo adjuvanticity of aluminum oxyhydroxide or 3M-052-AF, we immunized C57BL/6 mice with the tuberculosis vaccine antigen ID93 [18] without adjuvant or adjuvanted with either 3M-052-AF, aluminum oxyhydroxide, or 3M-052-AF bound to aluminum oxyhydroxide. Three weeks after the first immunization, mice receiving ID93 + 3M-052-AF-Alhydrogel® exhibited the highest serum titers of ID93-specific total IgG as well as IgG1 and IgG2c subtypes indicating that 3M-052-AF-Alhyrogel has unique adjuvant properties compared to either 3M-052-AF or Alhydrogel® alone (Fig. 6A). One month after the third immunization we assessed CD4 T cell responses by stimulating splenocytes with ID93 and measuring cytokine production in the presence of Brefeldin A by flow cytometry. Compared to mice immunized with ID93 alone, both ID93 + 3M-052-AF and ID93 + 3M-052-AF-Alhydrogel® immunized mice had greater frequencies of ID93-specific CD4 T cells expressing CD154. Importantly, only the mice immunized with ID93 + 3M-052-AF-Alhydrogel® exhibited TH1 cells that made IFN-γ, TNF, IL-2 and GM-CSF upon ID93 stimulation (Fig. 6B).

While the Th1-type response to 3M-052-AF-Alhydrogel® was generally consistent across multiple experiments, we note that there was more variability in biological response to 3M-052-AF (without Alhydrogel®), with some batches of 3M-052-AF inducing TH1 T cell adjuvant activity comparable to 3M-052-AF-Alhydrogel® (data not shown). Adsorption of 3M-052-AF to Alhydrogel® appeared to result in a more consistent biological activity between batches. Significant variation in biological response upon seemingly minor changes in formulation properties was also noted earlier for another aqueous nanosuspension, GLA-AF, from our lab [24]. For GLA-AF, we showed that systematically varying the phospholipid:TLR4 ligand ratio revealed a dramatic bi-phasic response in physicochemical as well as in vitro bioactivity assays [24]. Thus, if the phospholipid:3M-052 ratio employed in the present work is near such an inflection point, seemingly minor variations in the preparation or physical properties of the formulation could result in major changes in its biological activity. The effects of processing conditions, diluents, and vaccine antigen properties on the heterogeneous particle characteristics of 3M-052-AF also merit further investigation, which is ongoing in our lab.

**Fig. 7:**
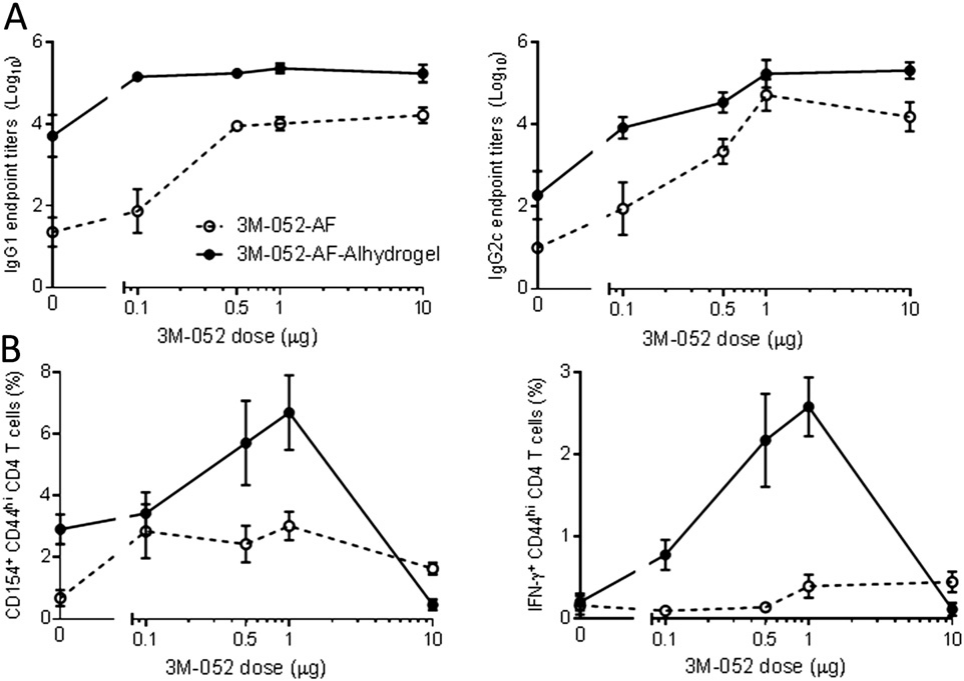
3M-052-AF and 3M052-AF-Alhydrogel® dose titration. C57BL/6 mice were immunized twice three weeks apart via intramuscular injection with ID93 (0.5 μg) alone or adjuvanted with 3M-052-AF (0.1, 0.5, 1, or 10 μg), Alhydrogel®, or 3M-052-AF (0.1, 0.5, 1, or 10 μg) bound to Alhydrogel®. (A) Three weeks after the first immunization ID93-specific IgG1, IgG2c, and total IgG serum endpoint titers were determined by ELISA. *N* = 5 mice/group. (B) One week after the final immunization splenocytes were restimulated with media or ID93 in the presence of Brefeldin A for 8 h and the frequency of cytokine producing CD4 T cells was determined by subtracting the media response from the ID93-specific response. Data are representative of two experiments with similar results with 5 animals per group. Mean ± s.e.m. is shown.

To determine whether binding 3M-052-AF to aluminum oxyhydroxide fundamentally changed its adjuvant activity or simply altered its bioavailability we examined the adjuvant activity of 3M-052-AF alone or bound to aluminum oxyhydroxide over a two log_10_ dose range. Three weeks after the first immunization with adjuvanted ID93 the Alum-adsorbed formulation of 3M-052-AF consistently elicited higher serum antibody titers across the entire dose range compared to either Alhydrogel® alone or the same dose range of 3M-052-AF (Fig. 7A). Similarly 3M-052-AF-Alhydrogel® demonstrated a bell shaped dose response for augmenting ID93-specific CD4 T cells with the peak response of the tested doses at 1 μg. These CD154 and IFN-γ responses were substantially higher than those elicited with ID93 adjuvanted with aluminum oxyhydroxide or 3M-052-AF at 0.1, 1, or 10 μg (Fig. 7B). Similar dose responses were observed for TNF and IL-2 producing CD4 T cells as well (data not shown). Based on this we conclude that binding 3M-052-AF to aluminum oxyhydroxide alters its adjuvant activity by some means other than simply changing the bioavailability over a 1 log_10_ range in either direction.

**Fig. 8:**
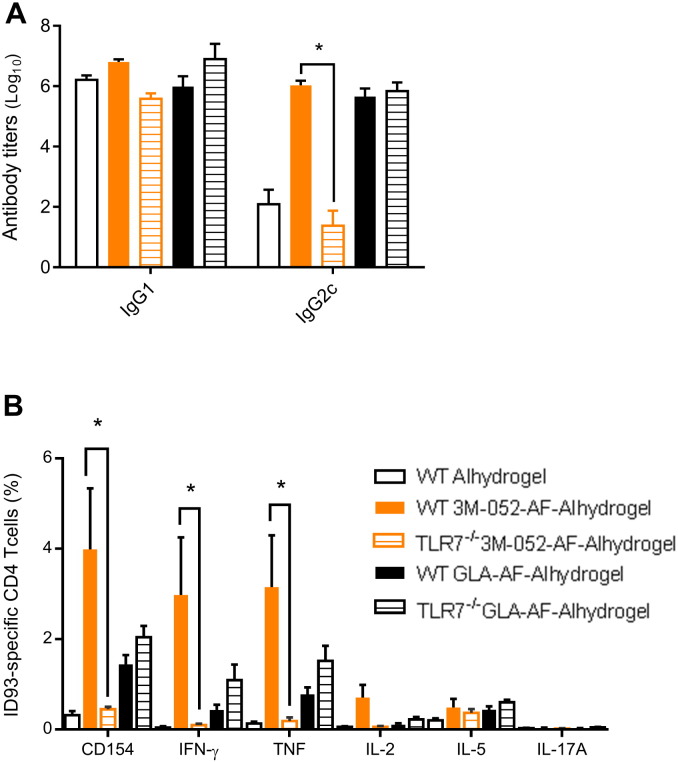
TLR7 is required for the TH1 inducing adjuvant activity of 3M-052-AF-Alhydrogel®. Wildtype C57BL/6 or B6.129S1-TLR7^tm1Flv^ (TLR7^-/-^) mice were immunized twice three weeks apart via intramuscular injection with ID93 (0.5 μg) adjuvanted with Alhydrogel®, 3M-052-AF (1 μg) bound to Alhydrogel®, or GLA-AF (5 μg) bound to Alhydrogel®. (A) ID93-specific IgG1 and IgG2c serum antibody titers were determined three weeks after the first immunization. (B) ID93-specific CD4 T cells were quantified following ex-vivo stimulation of splenocytes with ID93 one week after the second immunization. *N* = 5 mice/group. Data are representative of two experiments with similar results with 5 animals per group. Bars indicate mean ± s.e.m. *p < 0.05.

In vitro, 3M-052 (dissolved in DMSO) activates human TLR7 and TLR8 [16]. To determine whether these innate immune receptors are important for the in vivo adjuvant activity of 3M-052-AF-Alhydrogel® we compared the immune response of vaccinated wild type (WT) C57BL/6 mice and mice lacking TLR7 (C57BL/6 mice express a hypofunctional TLR8). As a control we also immunized C57BL/6 and TLR7-/- mice with ID93 adjuvanted with the TLR4 agonist adjuvant GLA-AF-Alhydrogel®. All of the immunized groups produced high titers of ID93-specific IgG1 antibodies, regardless of adjuvant or genotype (Fig. 8A). Both 3M-052-AF-Alhydrogel® and GLA-AF-Alhydrogel® also elicited high titers of IgG2c in C57Bl/6 mice compared to Alhydrogel® alone. In TLR7-/- mice the IgG2c induction was drastically reduced in animals immunized with ID93 + 3M-052-AF-Alhydrogel®, whereas the IgG2c response to ID93 + GLA-AF-Alhdyrogel® was not affected by TLR7 deficiency, confirming that TLR7 was necessary to recognize 3M-052, but not Alhydrogel® or GLA. ID93 + 3M-052-AF-Alhdyrogel® also elicited robust cellular responses in WT mice characterized by CD4 T cells capable of producing IFN-γ and TNF with very little IL-5 or IL-17A produced (markers of TH2 and TH17 immunity, respectively). However in TLR7-/- mice ID93 + 3M-052-AF-Alhydrogel® elicited only minor CD4 T cell responses to ID93 which were not substantially different in magnitude from the responses elicited by ID93 adjuvanted with Alhydrogel® alone in WT mice (Fig. 8B). Immunization of WT and TLR7-/- mice with ID93 + GLA-AF-Alhydrogel® elicited similar TH1 responses indicating that TLR7-/- mice are not impaired in their CD4 T cell response to vaccines with other TLR agonist containing adjuvants formulated on Alum. Therefore we conclude that similar to the in vitro findings, 3M-052-AF-Alhydrogel® requires TLR7 for its in vivo adjuvant activity, particularly in eliciting high frequencies of TH1 CD4 T cells and IgG2c switched antibody responses.

As noted above, we have previously employed the helper lipid approach to adsorb TLR4 ligands to aluminum oxyhydroxide. The otherwise insoluble TLR4 ligand GLA can be formulated as an aqueous suspension using a helper lipid, which can then be mixed with aluminum oxyhydroxide to allow for adsorption. However, in the case of GLA the agonist itself contains a phosphate group which promotes adsorption through ligand exchange. In the case of 3M-052, the agonist contains no phosphate group, thus adsorption due to ligand exchange must be entirely attributed to the helper lipid. Although in the present study ID93 + 3M-052-AF-Alhydrogel® appeared to induce a more potent TH1 response than ID93 + GLA-AF-Alhydrogel® (Fig. 8), further investigation along these lines is merited. Cellular location and distribution of TLR7/8 and TLR4 is significantly different and varies between species [13]. For example, TLR8 is considered refractory in mice; thus, an agonist such as 3M-052 may elicit altered or enhanced responses in humans or other species with functional TLR8. Such considerations, combined with the data presented here, indicate that an Alum-based TLR7/8 adjuvant formulation could provide a potent adjuvant formulation for TH1 responses in humans.

**Fig. 9:**
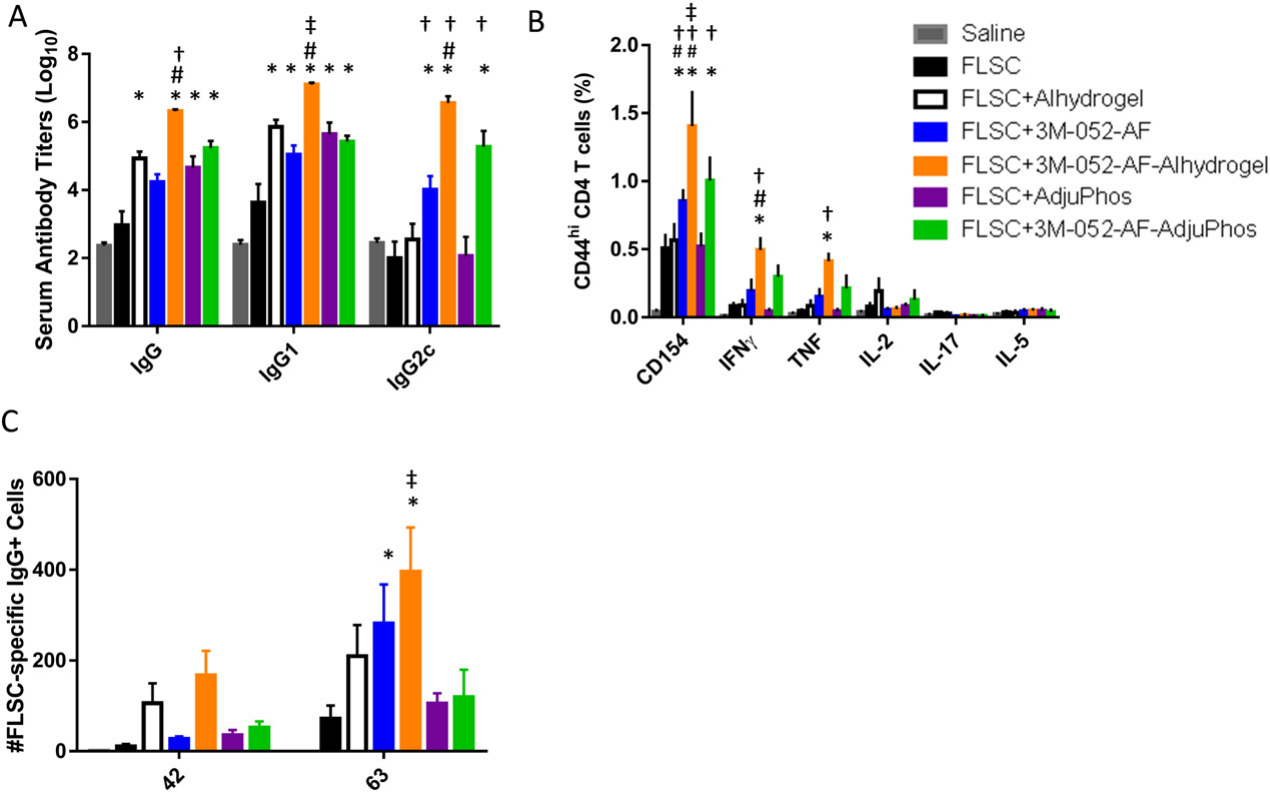
FLSC formulated with 3M-052-AF-Alhydrogel® induces enhanced antibody and TH1-type cellular responses compared to FLSC formulated with 3M-052-AF-AdjuPhos®, 3M-052-AF alone, or either type of Alum alone. C57BL/6 mice were immunized three times, three weeks apart via intramuscular injection with FLSC (10 μg) alone or adjuvanted with 3M-052-AF (1 μg), Alhydrogel®, AdjuPhos®, 3M-052-AF-Alhydrogel®, or 3M-052-AF-AdjuPhos®. (A) Three weeks after the first immunization FLSC-specific IgG1, IgG2c, and total IgG (IgG) serum endpoint titers were determined by ELISA. *N* = 5 mice/group, bars indicate mean + s.d. (B) One week after the second immunization splenocytes were restimulated with FLSC and the frequency of cytokine producing CD4 T cells was determined by flow cytometry. *N* = 5 mice/group, bars indicate mean ± s.d. (C) Three weeks after the second and third immunizations FLSC-specific bone marrow antibody-secreting cells were measured by ELISPOT. *N* = 5 mice/group, bars indicate mean ± s.e.m. *p < 0.05 vs. FLSC, #*p* < 0.05 vs. 3M-052-AF, †*p* < 0.05 vs. corresponding Alum (Alhydrogel® or AdjuPhos®), ‡*p* < 0.05 vs. 3M-052-AF-AdjuPhos®.

To test the in vivo adjuvant activity of 3M-052-AF with a different vaccine antigen as well as the effect of different aluminum salts, we immunized C57BL/6 mice with the HIV vaccine antigen FLSC [19] adjuvanted with either 3M-052-AF, aluminum oxyhydroxide, aluminum phosphate, or 3M-052-AF in combination with aluminum oxyhydroxide or aluminum phosphate. Three weeks after the first immunization, the most elevated serum IgG and IgG2c antibody responses were elicited by FLSC adjuvanted with 3M-052-AF-Alhydrogel® (Fig. 9A). Interestingly, 3M-052-AF-Alhydrogel® also produced the highest levels of mucosal IgG2c and antibody-secreting long lived plasma cells (Figs. 9C and S3). Moreover, the formulation containing 3M-052-AF and Alhydrogel® was the most potent inducer of IFNγ and TNF from CD4 + T cells (Fig. 9B), particularly one week after the prime immunization (Fig. S2). Overall, 3M-052-AF-Alhdyrogel® appeared to have more potent adjuvant activity in this model than 3M-052-AF-AdjuPhos®; however, since the FLSC antigen under these conditions adsorbs to aluminum oxyhydroxide (Alhydrogel®) but not aluminum phosphate (AdjuPhos®) (Fig. S3), reduced immunogenicity responses could be attributable to less optimal adsorption of both antigen and/or 3M-052-AF to aluminum phosphate.

**Fig. 10:**
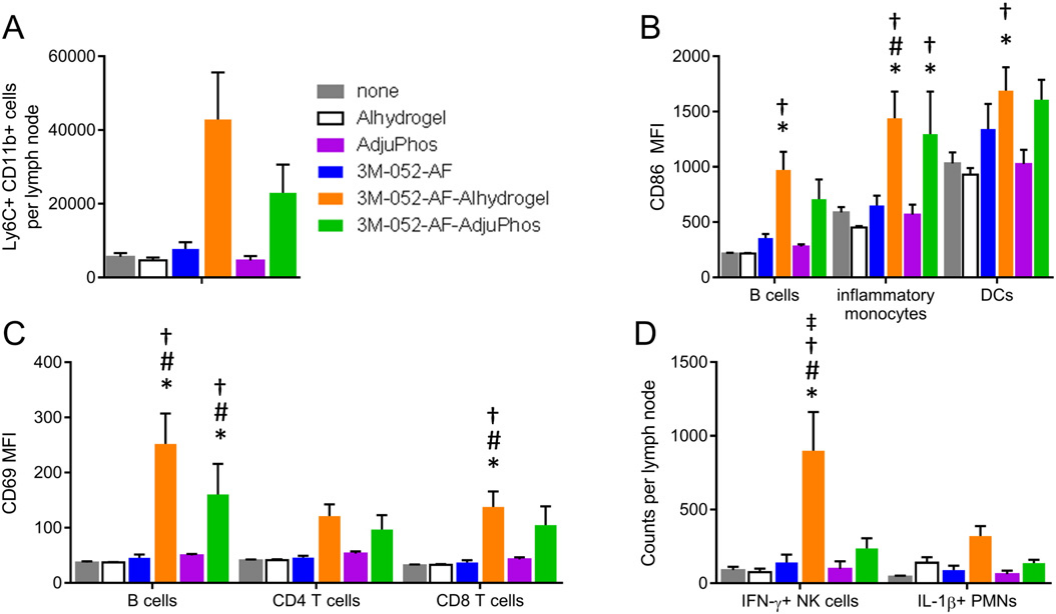
3M-052-AF and Alum synergize to augment innate responses upon immunization. Wildtype C57BL/6 mice were immunized intramuscularly with 3M-052-AF (1 μg), Alhydrogel®, AdjuPhos®, 3M-052-AF-Alhydrogel®, or 3M-052-AF-AdjuPhos®. 18 h later the draining inguinal lymph nodes were harvested and analyzed for (A) influx of CD11b + Ly6C + inflammatory monocytes, (B) expression of the costimulatory molecule CD86 on B cells, inflammatory monocytes or DCs (C) expression of CD69 on lymphocytes, and (D) expression of IFN-γ or IL-1b by NK cells and neutrophils, respectively. *N* = 5 mice/group. Data are representative of two experiments with similar results with 5 animals per group. Bars indicate mean + s.e.m. *p < 0.05 vs. none, #*p* < 0.05 vs. 3M-052-AF, †*p* < 0.05 vs. corresponding Alum (Alhydrogel® or AdjuPhos®), ‡*p* < 0.05 vs. 3M-052-AF-AdjuPhos®.

Induction of a robust adaptive immune response to vaccine antigens requires appropriate activation of the innate immune system to provide the necessary costimulatory and cytokine milieu. Thus we analyzed the innate immune responses in the draining lymph node that are altered by immunization with 3M-052-AF ± aluminum phosphate or aluminum oxyhydroxide. Although not statistically significant in all readouts, overall 3M-052-AF synergized with aluminum oxyhydroxide to enhance various innate immune responses (Fig. 10). Thus, 3M-052-AF appeared to synergize with aluminum oxyhydroxide and to a lesser extent aluminum phosphate to elicit a robust increase in the number of inflammatory monocytes (CD11b^+^ Ly6C^+^) in the draining lymph node 18 h after i.m. injection (Fig. 10A). Similarly 3M-052-AF and both Alum formulations augmented expression of the co-stimulatory molecule CD86 on APCs including B cells, monocytes and dendritic cells and transient activation of CD4 and CD8 T cells as well as B cells as indicated by CD69 expression (Fig. 10B and C). 3M-052-AF uniquely synergized with aluminum oxyhydroxide to augment number of NK cells expressing IFN-γ and showed trend of increased neutrophils producing IL-1 (Fig. 10D), both molecules important for the induction of robust TH1 responses with vaccine adjuvants [25]. This innate response to the synergy between 3M-052-AF and Alum likely creates the appropriate environment for the robust generation of adaptive immune responses to the vaccine antigens. The expansion of IFN-γ producing NK cells and IL-1 producing neutrophils correlates with the stronger adjuvant activity of 3M-052-AF + aluminum oxyhydroxide compared to the weaker responses induced by 3M-052-AF + aluminum phosphate.

3M-052-AF and aluminum hydroxide synergized to increase expression of the co-stimulatory molecule CD86 on APCs in the draining LN and to transiently activate lymphocytes to remain in the draining lymph node in an antigen-independent fashion. By activating APCs and trapping lymphocytes in the same draining LN this synergy creates an optimal environment for lymphocyte priming and expansion. Interestingly, innate responses including early production of IFN-γ and IL-1β which we found to be important for the adjuvanticity of the GLA-SE [25] were also most pronounced when 3M-052-AF was formulated with aluminum oxyhydroxide. This may suggest that these parameters could be useful universal signatures of effective adjuvant activity. Identification of such signatures would aid the rational development of new vaccine candidates.

## 4 Conclusion

In summary, we have developed a method to formulate lipid-based PRR ligands into aqueous nanosuspensions that can be made to adsorb to aluminum salts based on the properties of the helper lipid. The ability to develop Alum-compatible formulations of new PRR ligands may enable more rapid translation to the clinic since such formulations are analogous to the TLR4 ligand-Alum combination employed in Cervarix®, and aluminum salts are the most widely employed class of adjuvants in human vaccines, with a well-established safety and immunogenicity record.

## Appendix A Supplementary data

Supplementary material Image 1
