# Supplementary material for: Adsorption of a synthetic TLR7/8 ligand to aluminum oxyhydroxide for enhanced vaccine adjuvant activity: A formulation approach
Source: J Control Release. 2016 Dec 28;244(Pt A):98–107. doi: 10.1016/j.jconrel.2016.11.011 (PMC5176129; doi:10.1016/j.jconrel.2016.11.011)
Supplement: Supplementary file 1 — Supplementary material [file mmc1.docx]

**Supplementary Information to Fox et al. “Adsorption of a synthetic TLR7/8 ligand to aluminum oxyhydroxide for enhanced vaccine adjuvant activity: a formulation approach”**

This supplementary information contains supporting tables and figures containing adjuvant formulation development and stability data (Tables S1-S3 and Figure S1), additional in-vivo immunological readouts (Figure S2), and characterization of antigen-adjuvant interactions (Figure S3).

**Table S1. Effect of acyl chain and saturation on PG-based nanosuspension size and adsorption properties.**

| **Helper Lipid/Surfactant** | **Acyl Chain Length:Number of Unsaturated Bonds** | **Size (Z-Ave, nm)** | **PdI** | **Calculated Adsorption to Alhydrogel®** |
| --- | --- | --- | --- | --- |
| DLPG | 12:0 | 114 ± 9 | 0.79 ± 0.09 | 85 ± 2% |
| DMPG | 14:0 | 117 ± 47 | 0.25 ± 0.07 | 79 ± 0% |
| DPPG | 16:0 | 167 ± 13 | 0.38 ± 0.09 | 86 ± 1% |
| DSPG | 18:0 | 182 ± 1 | 0.44 ± 0.01 | 87 ± 1% |
| DOPG | 18:1 | 181 ± 9 | 0.52 ± 0.07 | 87 ± 0% |

Values represent average +/- s.d. of three measurements from the same sample for particle size and size polydispersity, or duplicate samples for the adsorption to Alhydrogel® experiment.

**Table S2. Stability of adsorption of 3M-052-AF over time and in the presence of co-adsorbed TLR ligands.**

| **TLR Ligand(s)** | **Aluminum Salt** | **TLR Agonist Conc. In Supernatant (µg/ml, T=0)** | **TLR Agonist Conc. In Supernatant (µg/ml, T=16wks)** |
| --- | --- | --- | --- |
| 3M-052-AF | - | 94 ± 1 | 74 ± 1 |
| GLA-AF | - | 93 ± 1 | 84 ± 4 |
| CpG ODN | - | 90 ± 3 | 81 ± 0 |
| 3M-052-AF/GLA-AF | - | 88 ± 1 / 94 ± 6 | 80 ± 2 / 89 ± 8 |
| 3M-052-AF/CpG ODN | - | 93 ± 1 / NM | 81 ± 1 / NM |
| 3M-052-AF | Alhydrogel® | 11 ± 1 | 10 ± 1 |
| GLA-AF | Alhydrogel® | <10 | <10 |
| CpG ODN | Alhydrogel® | <10 | <10 |
| 3M-052-AF/GLA-AF | Alhydrogel® | 11 ± 3 / <10 | <10 / <10 |
| 3M-052-AF/CpG ODN | Alhydrogel® | 11 ± 1 / <10 | <10 / <10 |

Notes: NM=not measured. 3M-052-AF contains DSPG whereas GLA-AF contains DPPC. CpG ODN is soluble and thus contains no helper lipid. Unbound TLR ligands were assayed by UV absorbance (3M-052, CpG) or HPLC with charged aerosol detection (GLA). Values represent average +/- s.d. of duplicate samples.

**Table S3. Effect of concentration of saline on adsorption of 3M-052-AF to Alhydrogel®.**

| **Saline Conc. (mM)** | **Aluminum Salt** | **3M-052 Conc. in Supernatant (µg/ml)** | **Adsorption to Alhydrogel®** |
| --- | --- | --- | --- |
| 60 | - | 79 ± 1 | - |
| 60 | Alhydrogel® | <10 | ≥87% |
| 120 | - | 61 ± 3 | - |
| 120 | Alhydrogel® | <10 | ≥84% |
| 240 | - | 57 ± 9 | - |
| 240 | Alhydrogel® | <10 | ≥82% |
| 480 | - | 51 ± 10 | - |
| 480 | Alhydrogel® | <10 | ≥80% |

Samples were centrifuged for 10 s at 2000 x g. Values represent average +/- s.d. of duplicate samples.


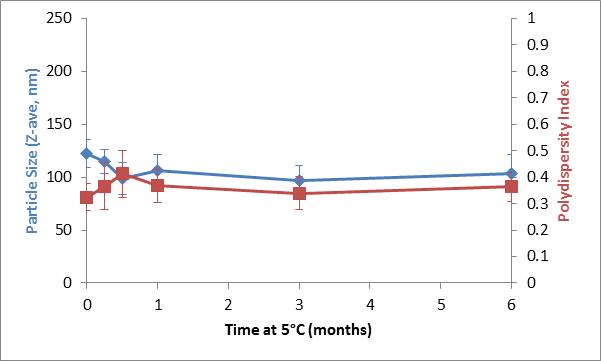


**Figure S1. 3M-052-DSPG (3M-052-AF) nanosuspension particle size and size polydispersity over 6 months (n=6 batches, average +/- s.d. is shown).**

**Figure S2. FLSC formulated with 3M-052-AF-Alhydrogel® induces enhanced vaginal and TH1-type cellular responses.** C57BL/6 mice were immunized three times, three weeks apart via intramuscular injection with FLSC (10 µg) alone or adjuvanted with 3M-052-AF (1 µg), Alhydrogel®, AdjuPhos®, 3M-052-AF-Alhydrogel®, or 3M-052-AF-AdjuPhos®. (A) Three weeks after the third immunization FLSC-specific IgG1, IgG2c, and total IgG vaginal lavage endpoint titers were determined by ELISA. N=9-10 mice/group, bars indicate mean +/- s.d. (B) One week following the first and second immunizations, splenocytes were restimulated with FLSC1 and the frequency of cytokine producing CD4 T cells was determined by flow cytometry. N=5 mice/group, bars indicate mean +/- s.e.m. *p<0.05 vs. FLSC, #p<0.05 vs. 3M-052-AF, †p<0.05 vs. corresponding Alum (Alhydrogel® or AdjuPhos®), ‡p<0.05 vs. 3M-052-AF-AdjuPhos®.


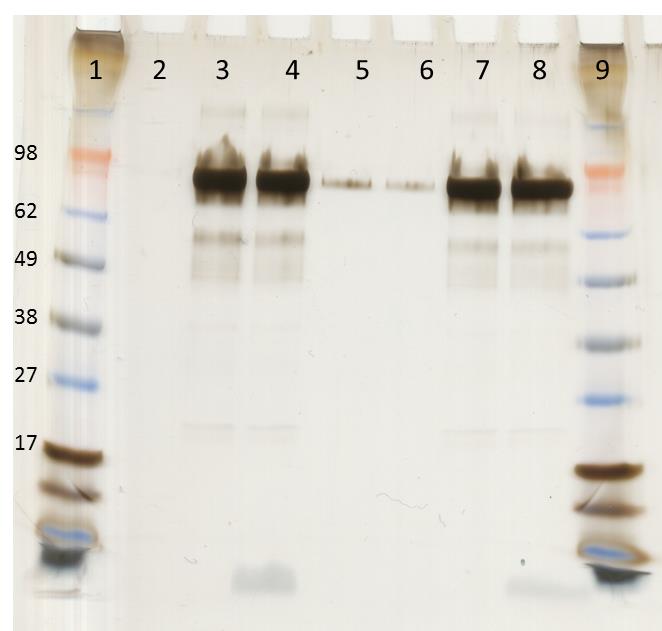


**Figure S3. FLSC binds to Alhdyrogel® but not to AdjuPhos®.** FLSC (100 µg/ml) was mixed with saline, 3M-052-AF (10 µg/ml), and/or different types of Alum (aluminum oxyhydroxide or aluminum phosphate, each at 1 mg/ml aluminum). The mixture was analyzed by silver-stain SDS-PAGE. Loss of protein band intensity indicates the protein is bound to aluminum. Lane 1: MW marker; Lane 2: saline; Lane 3: FLSC; Lane 4: FLSC+3M-052-AF; Lane 5: FLSC+Alhydrogel®; Lane 6: FLSC+3M-052-AF-Alhydrogel®; Lane 7: FLSC+AdjuPhos®; Lane 8: FLSC+3M-052-AF-AdjuPhos®; Lane 9: MW marker.
